# In vitro shear bond strength evaluation of resin cements between zirconia and titanium

**DOI:** 10.1038/s41598-025-10658-y

**Published:** 2025-07-16

**Authors:** Guanyu Su, Shujun Wang, Chuhan Zhang, Yiyang Tang, Mengting Wei, Ali Goudarzi, Lipeng Jiang, Jiale Fu

**Affiliations:** 1 School and Hospital of Stomatology, China Medical University, Shenyang, China; 2 Department of Radiation Oncology, The First Affiliated Hospital of Jinzhou Medical University, Jinzhou, China; 3 Department of Dental Materials Science, Digital Prosthodontic Center, School and Hospital of Stomatology, China Medical University, Shenyang, China

**Keywords:** Shear bond strength (SBS), Zirconia, Titanium, Resin cement, Storage condition, Medical research, Materials science

## Abstract

This study aimed to evaluate the Shear Bond Strength (SBS) and durability of different resin cements used for cement-retained restorations between zirconia crowns and titanium abutments. A total of 36 zirconia cubes and 324 titanium cylinders were employed in this study. Accordingly, 6 groups were assigned according to the used resin cement: three self-etching types (RU, AI, GMP), two self-adhesive types (IS, GM) and one unfilled type (SB). Each group was further divided into 3 sub-group based on storage condition (*n* = 10): 24-hour water storage at 37 °C, 5,000 thermocycles, and 10,000 thermocycles respectively. SBS testing employed a universal testing machine to quantify the force required for material fracture at the interface, followed by failure mode analysis. Two-way ANOVA showed significant influences of storage conditions and bonding agents on SBS values (*p* < 0.05), except for IS and GMP (*p* > 0.05). The SBS values of AI and RU showed a downward trend, while SB and GM showed an upward trend after 10,000 thermocycles. The SBS values of GM and GMP were significantly different in the 24-hour water storage but not after thermocycles. Within the limitations of the current study and based on the findings, the following can be concluded: (1) SBS was significantly influenced by both bonding agent type and storage condition; (2) Bonding systems containing HEMA and 10-MDP exhibited high initial strength but declined significantly after thermocycling; (3) IS, GM, and GMP demonstrated stable bond durability.

## Introduction

Implant treatments are now primarily replacing the traditional fixed dentures, especially in cases of single-tooth loss. Titanium abutments have become the preferred clinical choice due to their excellent mechanical properties and biocompatibility^1–4^. When combined with zirconia crowns that offer both high strength and pleasing aesthetics, this combination achieves optimal restorative outcomes^5–7^.

An implant restoration is traditionally classifyed into two types: cement-retained and screw-retained^8,9^. In clinical work, cement-retained restorations are easier to operate and achieve passive positioning compared to screw-retained restorations^10^. Moreover, without a screw hole on the surface of the restoration, the occlusal force is evenly distributed, making the restoration more aesthetically pleasing^11^. Patients with insufficient vertical space often result in a non-ideal retention form (abutment height < 5 mm), which cannot guarantee the durability of adhesion for the crown prosthesis^12^. Therefore, the longevity of restorations depends on adequate long-term bonding between the zirconia crowns and the titanium abutments^13–15^. Although extensive research has been conducted on zirconia bonding methods, the most suitable bonding protocol for the titanium abutment and the zirconia crown remains a matter of controversy^16–22^.

Since zirconia is chemically inert, it is difficult for untreated zirconia to bond to other materials^23^. Applying hydrofluoric acid etching and silane coupling agents for surface treatment is ineffective for zirconia due to its lack of silica components^24^. Sandblasting has been claimed to be the most effective pretreatment protocol for titanium and zirconia^25^. Air abrasion increases the bond surface area by roughening the material’s surface, thereby enhancing the bond strength between the resin cement and the oxide ceramics while also improving the wetting kinetics of the adhesives and removing organic contaminants from the ceramic surface^26,27^.

With the continuous optimization of adhesive components, self-etching resin cement and self-adhesive resin cement have evolved as innovative and user-friendly bonding agent types for attaching zirconia crowns to titanium abutments^28,29^. Simultaneously, it has been demonstrated that utilizing an adhesive monomer based on 10-methacryloyloxydecyl dihydrogen phosphate (10-MDP) can enhance the bond strength between zirconia and titanium^30–32^. While 10-MDP-containing self-adhesive cements were developed to eliminate separate 10-MDP priming for zirconia bonding, the potential additional benefit of combining these cements with additional 10-MDP primers requires further investigation^33,34^. Moreover, the Super-Bond C&B resin, which is based on methyl methacrylate (MMA), initiated by tri-n-butyl borane (TBB), and contains 4-methacryloyloxyethyl trimellitate anhydride (4-META) as the adhesive functional monomer, has been considered as a reliable agent for a long time^35,36^.

Accordingly, the present study aimed to evaluate the bonding performance of different types of adhesives between zirconia and titanium under different storage conditions. The null hypotheses tested were that: (1) Storage conditions do not significantly affect SBS; (2) There are no differences in bonding performance among the tested resin cements; (3) There is no significant difference in SBS between GM (self-adhesive mode) and GMP (self-etching mode).

## Materials and methods

A total of 36 zirconia cubes (with a volume of 2 cm × 2 cm × 2 cm) and 324 titanium cylinders (with a diameter of 4 mm) were employed in this study. The chemical compositions of the materials used in the present study are listed in Table 1.

**Table 1:** Materials used in the study. Bis-GMA: bisphenol A glycerolate dimethacrylate, HEMA: hydroxyethyl methacrylate, 10-MDP: 10-methacryloxy decyldihydrogen phosphate, TBB: tributylboron, 4-META: 4-methacryloxyethytrimethic anhydride, MMA: Methyl methacrylate, PMMA: polymethyl methacrylate, UDMA: urethane dimethacrylate, γ-MPTS: γ-methacryloxypropyltrimethoxysilane, MDTP: Methacryloyloxydecyl dihydrogen thiophosphate, TEG-DMA: triethyleneglycol dimethacrylate, PAA: polyalkenoic acid copolymer.

Code	Materials (Lot NO.)	Manufacturer	Composition	Application procedure
IS	Multilink speed (NE79372)	Ivoclar Vivadent (Liechtenstein)	Monomer matrix: dimethacrylates, acidic monomers Inorganic fillers: barium glass, ytterbium trifluoride, co-polymer, highly dispersed silicon dioxide Additional contents: initiators, stabilizers, color pigments	1. Directly apply Ivoclar Multilink Speed on the titanium. 2. Apply cement on the titanium. 3. Place the titanium on the zirconia and remove the excess cement. 4.Light cure for 10 s.
AI	Aidite Adhesive (20210515) Aidite Cement (20221210)	Aidite (China)	Bis-GMA, HEMA, 10-MDP, silica Bis-GMA, glass powder, silica	1. Apply Aidite adhesive to both surfaces and rub it in for 5 s. 2. After blowing and light-curing, place the titanium with Adite cement onto the zirconia immediately. 3. Excess adhesive was removed around the titanium. 4. Light cure for 10 s.
RU	Rely X Ultimate (9037035)	3 M ESPE (USA)	Base paste: methacrylate monomers, radiopaque, silanated fillers, initiator components, stabilizers, rheological additives Catalyst paste: methacrylate monomers, radiopaque alkaline (basic) fillers, initiator components, stabilizers, pigments, rheological additives, fluorescence dye, dual-cure activator for single bond universal adhesive	1. The cementation surfaces were treated with SBU. 2. Blow to thin with gentle air and light cure for 15 s–10 s separately. 3. Place mixed Rely X Ultimate on titanium and bond it to zirconia immediately. 4. Remove excess adhesive around the bottom of the titanium, then light cure for 10 s.
SB	Super-Bond C&B (VR1, VF1FEV12, ET1, EV32)	Sun Medical CO LTD(Japan)	TBB, 4-META, MMA, PMMA, 65% phosphoric acid, 10% citric acid, 3% ferric trichloride	1. Mix the A and B liquids of liner M in a 1:1 ratio and apply the mixture to the bonding surfaces. 2. Use the cooled mixing plate to mix the initiator and monomer liquid in a 1:4 ratio to get the active liquid. 3. Add an appropriate amount of L-type Radiopaque to the active liquid and apply the adhesive to the titanium. 4. Bond the titanium cylinder to the surface of the zirconia and allow it to cure naturally.
GM	G-CEM ONE (2202182)	GC (Japan)	Paste A: fluoroaluminosilicate glass, UDMA, dimethacrylate, initiator, SiO2, 3-Methacryloxypropyltrimethoxysilane, stabilizer, pigment, 10-MDP, methyl ester, 2-Propenoic-3,3,3-d3 acid Paste B: SiO2, trimethoxysilane, 2-hydroxy-1,3-dimethacryloxypropane, MgO, 6-tert-butyl-2,4-xylenol, 2,6-di-tert-butyl-p-cresol, UDMA, EDTA disodium salt dehydrate, vanadyl acetylacetonate, ascorbic acid, camphorquinone, 10-MDP, 3-Methacryloxypropyltrimethoxysilane, 2-Propenoic-3,3,3-d3 acid, methyl ester, 12-Methacryloyldodeylphosphate, cumyl hydroperoxide	1. Mix and apply G-CEM ONE to the surface of the titanium cylinder, bonding it to the zirconia. 2. Then, the excess adhesive was removed with a micro-brush. 3. Light cure for 10 s.
GMP	G-Multi PRIMER (2207081)	GC (Japan)	Ethanol, γ-MPTS, 10-MDP, MDTP, Bis-GMA, TEG-DMA, Vinyl silane, 12-Methacryloyldodeylphosphate, 2-Propenoic-3,3,3-d3 acid, methyl ester, thiophenol monomer	1. Apply G-multi primer to bonding interfaces, then blow to a thin, uniform layer and cure for 10 s. 2. Apply G-CEM ONE on the titanium. 3. After the titanium was placed on the zirconia, the excess adhesive was removed with a micro-brush. 4. Light cure for 10 s.
SBU	Single Bond Universal (11220 A)	3 M ESPE (USA)	10-MDP, dimethacrylate resins, HEMA, PAA, filler, ethanol, water, initiators, silane	
	Ivoclean (Y49501)	Ivoclar Vivadent (Liechtenstein)	Zirconium oxide, water, polyethylene glycol, sodium hydroxide, pigments, additives	
	Zirconia ceramic cube (L2220228125)	UPCERA (China)		
Code	Materials (Lot NO.)	Manufacturer	Composition	Application procedure

### Surface Preparation

The bonding interfaces on the specimens were treated by airborne-particle abrasion using 50 μm alumina particles from 10 mm at a pressure of 2.8 bar for 40 s on the zirconia cube and 10 s on the titanium cylinder, respectively, in order to obtain a good bonding strength and prevent zirconia’s surface from microcracks and phase transformation at the same time^30,37–39^. After sandblasting, the bonding interfaces of the titanium and zirconia were cleaned with Ivoclean (Ivoclar Vivadent AG) for 5 s. The bonding surface of each specimen was then rinsed thoroughly with water for 15 s and gently air-dried.

### Experimental groups and operations

The zirconia and titanium specimens were divided into six groups, each containing 6 cubes and 54 cylinders, based on the bonding resin used. The six bonding agents used in the present study were as follows:

Group 1 (SB): Superbond C&B + Porcelain LinerM (control group).

Group 2 (IS): Ivoclar Multilink Speed (self-adhesive).

Group 3 (AI): Aidite Cement + Aidite adhesive (self-etching).

Group 4 (GM): G-CEM ONE (self-adhesive).

Group 5 (GMP): G-CEM ONE + G-Muti PRIMER (self-etching).

Group 6 (RU): Rely X Ultimate + Single Bond Universal (self-etching).

This experiment focused on three bonding agent types, including an inorganic-filler-free control group (SB) and two conventional bonding systems: a self-etching system (RU, AI, GMP) and a self-adhesive system (IS, GM). In each group, titanium specimens were positioned and cemented to zirconia cubes under a constant load of 10 N and then light-cured with an LED light-curing unit (Kerr Demi Plus; Kerr, CA, USA) for 10 s. The chemical composition and usage methods is shown in Table 1.

### SBS test

For each subgroup, one-third of the titanium cylinders were tested after being stored in a thermostat (Cole-Parmer, Vernon Hills, IL, USA) containing distilled water at 37 ºC for 24 h. Another one-third underwent testing following 5,000 thermocycles between 5 ºC and 55 ºC with a dwell time of 25 s and transfer time of 10 s per bath, using a thermocycling device (SD Mechatronik, Feldkirchen-Westerham, Germany). The remaining titanium cylinders were tested after undergoing 10,000 cycles of thermocycling prior to the SBS test.

**Fig. 1:**
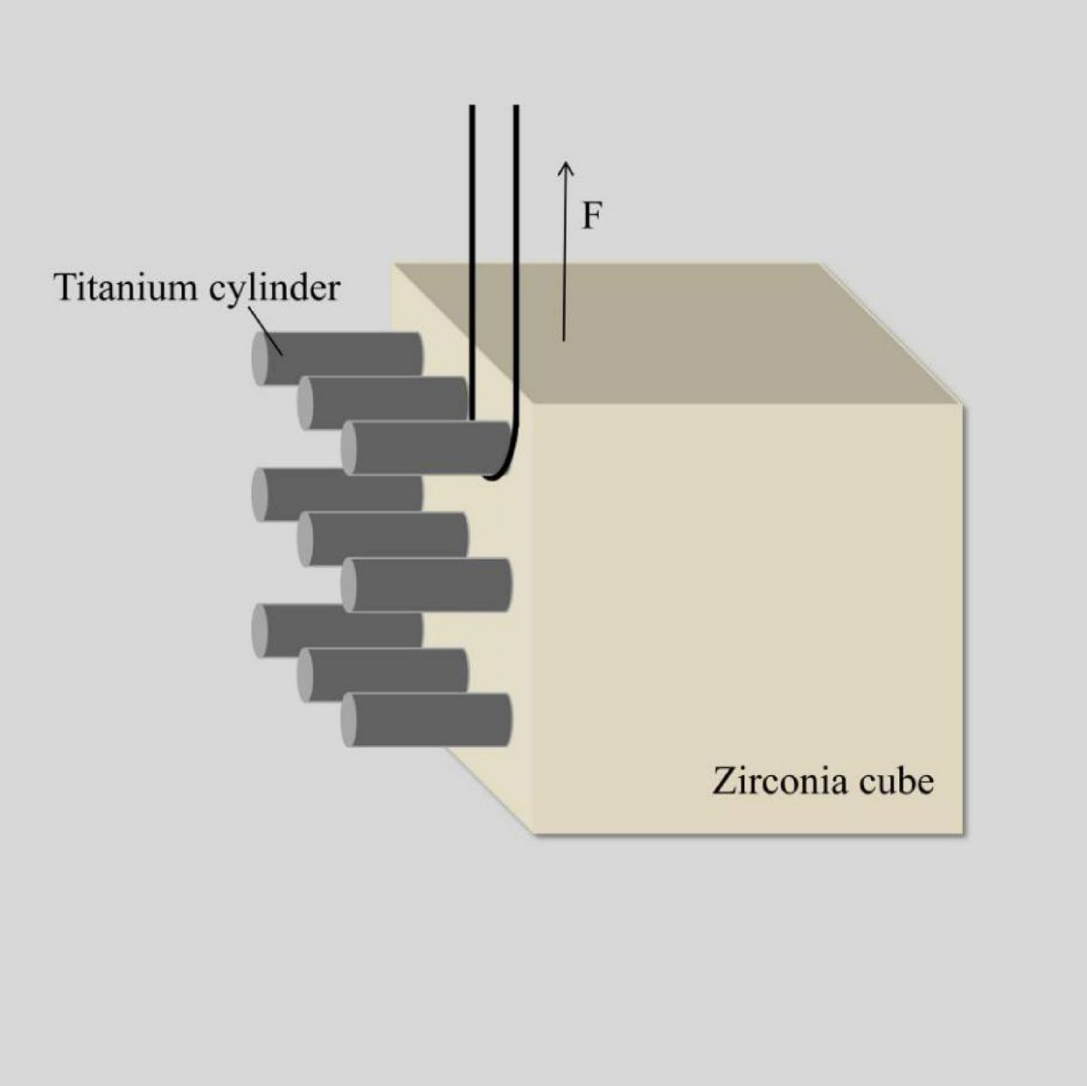
Diagram of specimen setting for SBS test. The SBS test was performed using a universal testing machine (WD-200 Weidu, Wenzhou, China) with a crosshead speed of 1 mm/min. A load was applied until the titanium cylinder debonded. SBS was calculated using the formula: P (MPa) = F (N)/S (mm²), where P is the SBS, F is the applied force, and S is the bonded surface area. The diagram illustrates the setup of the specimen during the SBS test.

The SBS test was performed using a universal testing machine (WD-200 Weidu, Wenzhou, China), with a load applied at a crosshead speed of 1 mm/min until the titanium cylinder debonded. SBS was calculated using the following formula: P (MPa) = F (N)/S (mm²). The specimen setting for the SBS test is shown in Fig. 1.

### Adhesive remnant index (ARI) scoring

**Fig. 2:**
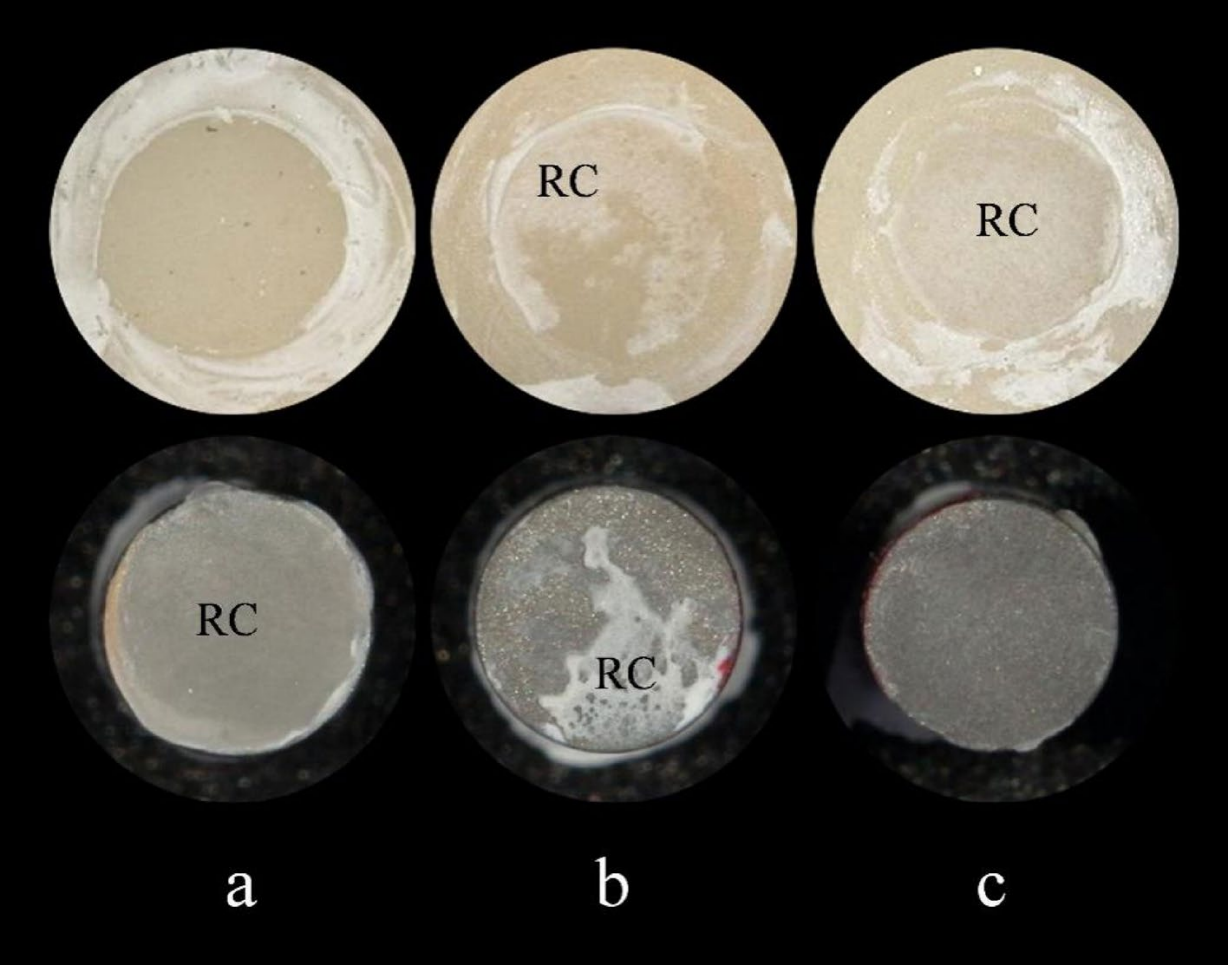
Representative images of different adhesive remnant index (ARI) scores. The left cylinder “**a**” represents Score 1 (I), no cement left on the zirconia surface (0%). The middle cylinder “**b**” represents Score 2 (II), partial cement left on the zirconia surface (0-100%, not counting 0 and 100%). The right cylinder “**c**” represents Score 3 (III), almost all cement left on the zirconia surface (100%). Label “RC” indicates the presence of resin cement.

An adhesive remnant index (ARI) system was employed to evaluate the cement residue on the zirconia. The scoring system consists of three levels, ranging from 1 to 3, with representative images for each level documented using a dental digital camera (EyeSpecial C-IV, Shofu, Kyoto, Japan) (Fig. 2). To reduce the likelihood of judgment errors, five independent observers scored all images, and the majority score was designated as the final rating. The scoring scale is presented below:

Score 1 (I): No cement left on the zirconia surface (0%).

Score 2 (II): Partial cement left on the zirconia surface (0-100%, not including 0 and 100%).

Score 3 (III): Almost all cement left on the zirconia surface (100%).

### Statistical analysis

In this study, the four highest and four lowest data points were removed, and then 10 remaining data points were selected for analysis (*n* = 10). A statistical analysis was performed with SPSS version 27.0 (SPSS, Chicago, IL, USA). Levene’s test and the Kruskal-Wallis test were performed, and the results indicate that the variance is homogeneous and the data follow a normal distribution. Then, a Two-way ANOVA examined the effect of different bonding materials and storage conditions on the SBS of the specimens (α = 0.05, 1-β = 0.8). In each group, multiple comparisons were conducted using either the Tukey HSD test or the Games-Howell test, depending on whether the assumption of homogeneity of variance was met (*P* ≤ 0.05). Additionally, another Two-way ANOVA was performed to compare the two etching modes of the same brand bonding agent (GC, Tokyo, Japan), followed by the Tukey HSD test (*P* ≤ 0.05).

## Results

**Fig. 3:**
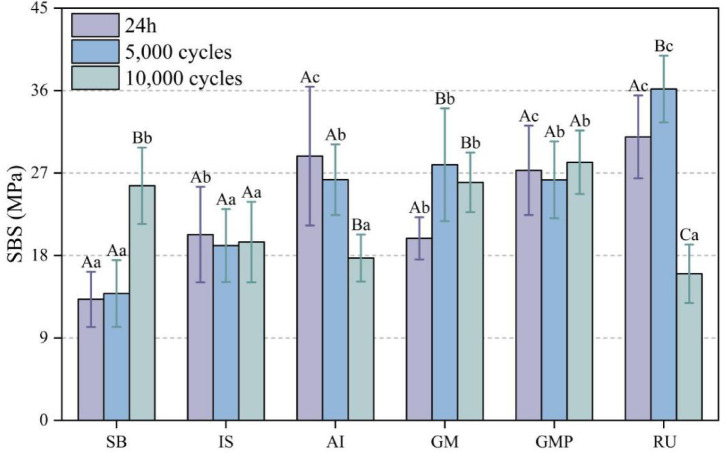
The mean SBS values (MPa) of all six resin cements under three storage conditions (mean ± SD). Six bonding agents are Group 1 (SB): Superbond C&B + Porcelain LinerM (control group), Group 2 (IS): Ivoclar Multilink Speed (self-adhesive), Group 3 (AI): Aidite Cement + Aidite adhesive (self-etching), Group 4 (GM): G-CEM ONE (self-adhesive), Group 5 (GMP): G-CEM ONE + G-Muti PRIMER (self-etching), Group 6 (RU): Rely X Ultimate + Single Bond Universal (self-etching)Three storage conditions are after 24 h, 5,000 thermal cycles, and 10,000 thermal cycles. The same capital letters indicate no significant differences among storage conditions for each resin cement agent  (*p* > 0.05). The same lowercase letters indicate no significant differences among resin cement agents for each storage condition (*p* > 0.05).

The mean SBS values (mean ± SD) of the six bonding agents under three different storage conditions are presented in Table 2; Fig. 3. The SBS values of IS (20.29 ± 5.21/19.09 ± 3.98/19.47 ± 4.39 MPa) and GMP (27.30 ± 4.88/26.26 ± 4.19/28.18 ± 3.47 MPa) did not show statistically significant differences across the three storage conditions (*p* > 0.05). After undergoing 5,000 and 10,000 thermocycles, the SBS values of AI (28.85 ± 7.57/26.28 ± 3.85/17.73 ± 2.57 MPa) and RU (30.95 ± 4.53/36.18 ± 3.64/16.01 ± 3.20 MPa) exhibited a decreasing trend. Conversely, the SBS values of SB (13.22 ± 3.01/13.86 ± 3.64/25.62 ± 4.17 MPa) and GM (19.88 ± 2.30/27.91 ± 6.16/25.99 ± 3.2 MPa) showed an upward trend. The SBS values for the GM and GMP were found to differ significantly after being stored at 37 °C in a water bath for 24 h. However, no significant difference was observed between the GM and GMP following 5,000 and 10,000 thermocycles. Two-way ANOVA revealed a significant influence of storage conditions (*p* < 0.05, F = 29.233) and resin cement types (*p* < 0.05, F = 6.318) on SBS values. Furthermore, the interaction among these two factors was also significant (*p* < 0.05, F = 21.234) (Table 3).

**Table 2:** The mean SBS values (MPa) of all six resin cements under three storage conditions (mean ± SD). The same uppercase letters indicate no significant difference in SBS within the same group under different storage conditions (*p* > 0.05). The same lowercase letters indicate no significant difference in SBS among different groups under the same storage condition (*p* > 0.05).

	SB	IS	AI	GM	GMP	RU
24 h	13.22 ± 3.01^A, a^	20.29 ± 5.21^A, b^	28.85 ± 7.57^A, c^	19.88 ± 2.30^A, b^	27.30 ± 4.88^A, c^	30.95 ± 4.53^A, c^
5,000 cycles	13.86 ± 3.64^A, a^	19.09 ± 3.98^A, a^	26.28 ± 3.85^A, b^	27.91 ± 6.16^B, b^	26.26 ± 4.19^A, b^	36.18 ± 3.64^B, c^
10,000 cycles	25.62 ± 4.17^B, a^	19.47 ± 4.39^A, b^	17.73 ± 2.57^B, b^	25.99 ± 3.25^B, a^	28.18 ± 3.47^A, a^	16.01 ± 3.20^C, b^

**Table 3:** Two-way ANOVA results of all bonding agents in different storage conditions.

Source	df	Mean square	F	*p*
Corrected model	17	396.452	21.832	0.000
Intercept	1	99435.082	5475.705	0.000
Storage conditions	5	530.853	29.233	0.015
Bonding agents	2	114.722	6.318	0.002
Storage conditions* Bonding agents	10	385.598	21.234	0.000

Table 4 shows the mean SBS values of the two bonding agent types. In conclusion, the self-etching bonding method provided higher initial bond strength and maintained this advantage after 5,000 thermocycles. However, after 10,000 cycles, the SBS of both types became comparable, with no significant difference observed. These findings suggest that while self-etching may be preferable for initial bond strength, the choice of bonding agent types may need to be reconsidered in the context of long-term thermal stress. Two-way ANOVA demonstrated that the SBS values of two etching modes of the same GC-brand bonding agent were significantly influenced by both the storage condition (*p* < 0.05, F = 4.553) and the etching mode (*p* < 0.05, F = 5.884), with a significant interaction between the two factors (*p* < 0.05, F = 5.790) (Tables 5 and 6).

**Table 4:** The mean SBS values (MPa) of the two bonding agent types. The same lowercase letters indicate no significant differences (*p* > 0.05).

	Self-Etching (RU, AI, GMP)	Self-Adhesive (IS, GM)
24 h	29.03 ± 5.81^a^	20.09 ± 3.92^b^
5,000 cycles	29.57 ± 5.95^a^	23.50 ± 6.78^b^
10,000 cycles	20.64 ± 6.23^b^	22.73 ± 5.03^b^

**Table 5:** The mean SBS values (MPa) of the GM and GMP groups. The same uppercase letters indicate no significant difference in SBS within the same group under different storage conditions (*p* > 0.05). The same lowercase letters indicate no significant difference in SBS among different groups under the same storage condition (*p* > 0.05).

	GM (Self-Adhesive)	GMP (Self-Etching)
24 h	19.88 ± 2.30^A, a^	27.30 ± 4.88^A, b^
5,000 cycles	27.91 ± 6.16^B, a^	26.26 ± 4.19^A, a^
10,000 cycles	25.99 ± 3.25^B, a^	28.18 ± 3.47^A, a^

**Table 6:** Two-way ANOVA result of the GM and GMP groups.

Source	df	Mean square	F	*p*
Corrected model	5	95.006	5.314	0.000
Intercept	1	40312.339	2254.705	0.000
Storage conditions	2	81.399	4.553	0.015
Processing mode	1	105.205	5.884	0.019
Storage conditions* Processing conditions	2	103.514	5.790	0.005

The ARI scores of each group are shown in Table 7. The ARI scores indicate that the adhesion quality of the groups was generally maintained over time, with a majority of samples achieving a score of 2 across all testing conditions. The IS and RU groups showed particularly high initial adhesion, with the RU group maintaining a consistently high score throughout the testing periods. The AI group, in contrast, did not achieve a Score 1 at any time point.

**Table 7:** ARI scores of each group. Provide the adhesion rating index (ARI) scores for each experimental group following the testing conditions of 24 h, 5,000 cycles, and 10,000 cycles. The ARI scores are used to quantify the degree of cement remaining on the zirconia surface after debonding, with score 1 (I): no cement left on the zirconia surface (0%); score 2 (II): partial cement left on the zirconia surface (0-100%, not including 0 and 100%); score 3 (III): almost all cement left on the zirconia surface (100%).

Group tested	ARI scores (24 h/5,000 cycles/10,000 cycles)		
	I	II	III
SB	2/0/0	8/8/10	0/2/0
IS	3/0/0	7/5/10	0/5/0
AI	0/0/0	10/10/9	0/0/1
GM	0/0/2	10/8/8	0/2/0
GMP	0/0/2	10/6/8	0/4/0
RU	8/0/0	2/10/10	0/0/0

## Discussion

In this experiment, an artificial aging process was performed to verify the long-term effect of adhesion. To simulate the oral environment, thermocycles were performed at temperatures between 5 and 55 °C, with 5,000 and 10,000 thermocycles representing 0.5-year and 1-year clinical periods, respectively^40,41^. The results indicated that SBS values differed across various systems under different storage conditions; consequently, the first null hypothesis, stating that storage conditions do not significantly affect SBS, was rejected.

Interestingly, after 5,000 thermocycles, the SBS of the RU and GM groups was significantly higher than that of the AI group. This enhancement is likely due to heat exposure from the 55 °C water bath, which facilitates in the resinous polymerization of the cements, as well as the stress relaxation that occurs following water absorption. Previous reports have linked the initial increase in bond strength to post-polymerization of the cements^42,43^.

Thermocycling generally reduced the resin bond; however, in the SB specimens, significantly higher SBS was observed after 10,000 thermocycles compared to both the control group and the group subjected to 5000 cycles, between which no statistically significant difference was found. These results are in line with the findings observed and reported by Shinagawa et al., who suggested that SB’s initially low bond strengths may be attributed to its self-curing nature, as the polymerization speed may not have sufficiently progressed sufficiently to provide good bonding at the earliest period^44^. Additionally, reasons for this increase may include the stable physical properties of SB, such as water absorption and solubility, its special composition, its pH-neutralization behavior, and the mature bonding between surfaces that favors bonding strength^45^. TBB acts as a polymerization initiator with enhanced polymerization activity upon exposure to water and air^46^.This increased reactivity is attributed to the formation of butyl and butoxy radicals through coordination between its boron atom and oxygen, with the butyl radical initiating polymerization^46,47^.

**Fig. 4:**
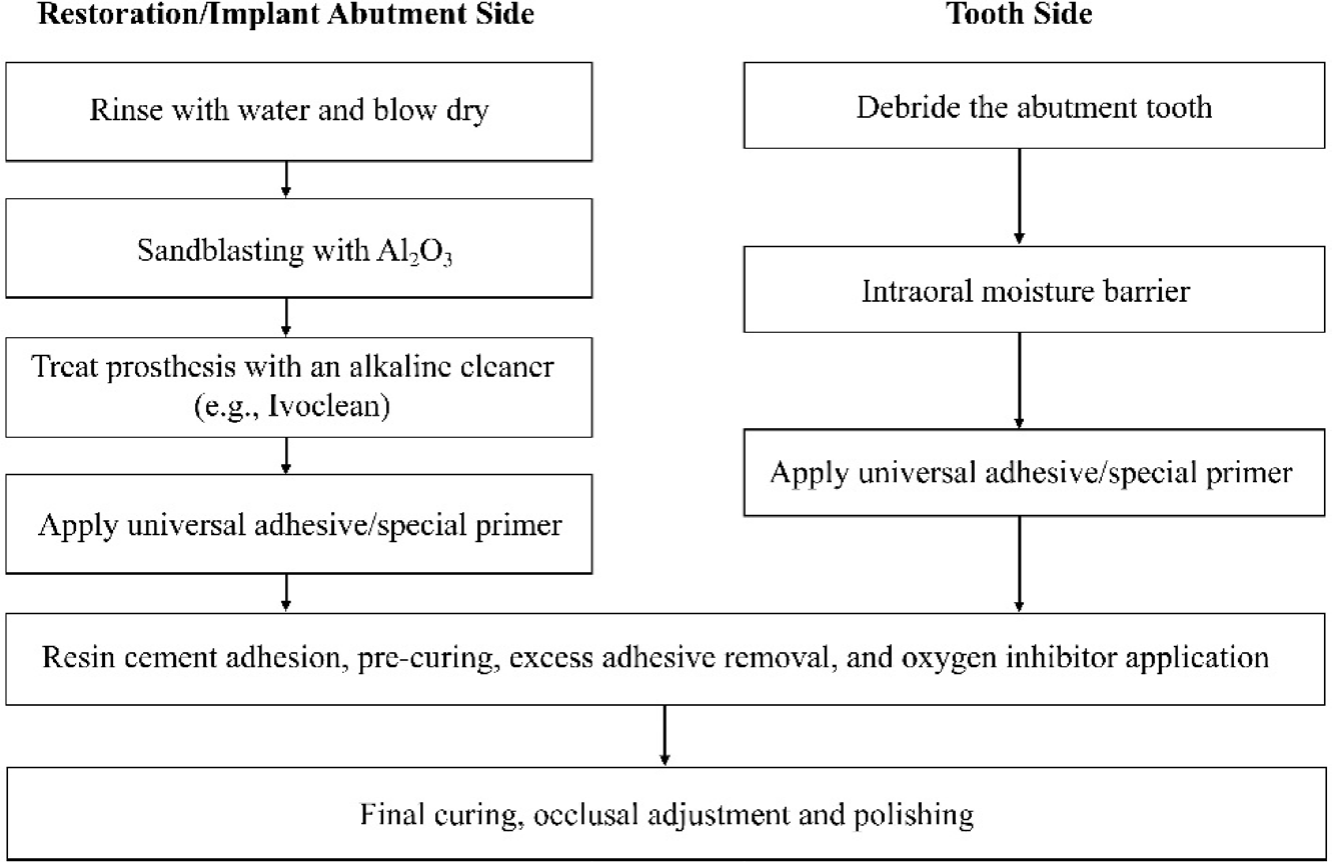
Clinically recommended procedure. This flowchart illustrates the recommended clinical steps for adhesive cementation of restorations or implant abutments. The left side outlines the surface treatment of the restoration/implant abutment, including rinsing, drying, Al_2_O_3_ sandblasting, alkaline cleaning (e.g., Ivoclean), and application of universal adhesive or special primer. The right side describes the preparation of the tooth, including debridement, moisture control, and universal adhesive or special primer application. Both paths converge into resin cement adhesion, pre-curing, excess adhesive removal, oxygen inhibitor application, followed by final curing, occlusal adjustment, and polishing.

Resin cements are generally considered more suitable than conventional cements for cementation between the ceramic crowns and the titanium abutments due to their improved retention, reduced microleakage, high tension strength, and acceptable clinical bonding performance^19,48–51^. Various kinds of phosphate monomers, such as 10-MDP, 6-methacryloyloxyhexyl phosphonoacetate (6-MHPA), 4-META, glycerol phosphate dimethacrylate (GPDM), and dipentaerythritol penta-acrylate monophosphate (PENTA), all of which contribute to the enhancement of bond strength of zirconia by the formation of P-O-Zr bonds^30,52–55^. Currently, 10-MDP is the most widely used. Several studies have reported that titanium alloys also require Al_2_O_3_ particle air abrasion with phosphate monomers to strengthen the bond^56,57^. Air abrasion may also increase irregularities, surface energy, and wettability, whereas phosphate monomers could lead to the formation of P-O-Ti bonds^58^. Figure 4 illustrates recommended clinical operating procedures tailored to the specific characteristics of different bonding interfaces. Following this workflow may help ensure optimal bond durability and improve the prospects for long-term clinical success. In this experiment, despite the consistent surface treatment methods used among all groups, significant differences in SBS were observed, thereby rejecting the second null hypothesis that there are no differences in bonding performance among the tested resin cements.

A surface free of contaminants is necessary for adhesion operation^59,60^. Saliva contamination occurs during clinical procedures for adjusting zirconia restorations in the oral environment. It was verified that the biofilm formed on the inner surface could be effectively removed, and the zirconia could be reactivated by an alkaline cleaner^61^. Hence, Ivoclean was employed to simulate the clinical situation in the present study.

In the experiment, AI, as a self-etching resin cement system, required the combined use of an adhesive and resin cement. Both AI and RU exhibited relatively high initial SBS values, which declined after long-term thermocycling. These findings suggest that the comparable long-term performance of AI and RU after 10,000 thermocycles may be attributed to the presence of HEMA and 10-MDP in both bonding systems. HEMA, a hydrophilic resin monomer, facilitates the formation of a strong initial bond but absorbs water over time, resulting in resin softening and reduced long-term performance^51^. Additionally, the primary limitation of 10-MDP is its susceptibility to hydrolytic degradation, resulting in a gradual decline in adhesion over time^62,63^.

Clinically, durable resin–zirconia/titanium bonds are crucial for long-term success in implant-supported prostheses. SB, IS, GM, and GMP indicated better durability in the present study due to their stable (IS and GMP) or increased (GM) SBS after 10,000 times thermocycling (Fig. 3). It is noteworthy that there was a significant difference in the SBS values of GM and GMP after 24 h storage. However, there was no significant difference after 5,000 and 10,000 thermocycles. The initial SBS values of GM after 37 ℃ water storage were significantly lower than those of GMP due to the extra application of phosphate monomers containing G-Multi PRIMER on both bonding surfaces; therefore, the SBS was improved in GMP. In the experiment, the use of primer significantly influenced SBS during the initial cementation stage (Table 5), and Two-way ANOVA showed that the SBS of GM and GMP was influenced by storage conditions and processing mode (Table 6). Thus, the third null hypothesis that there is no significant difference in SBS between GM (self-adhesive mode) and GMP (self-etching mode) was partially rejected. When resin cements can be used in both self-etching and self-adhesive modes, the use of a primer is recommended to ensure initial bond strength.

However, after undergoing thermal cycling, heating facilitated the curing reaction in the GM group, leading to an eventual equilibration of their SBS values. These findings are consistent with those of Ramos et al., indicating that this simplified zirconia-bonding protocol is a viable alternative to the primer/composite cement combination^64^. Suggesting that in clinical practice, G-CEM ONE can optionally be used without G-Multi PRIMER, maintaining stable performance regardless of the application mode. This result is consistent with the fracture mode analysis.

In the present study, while the mean SBS value of three self-etching resin cements was significantly higher than two self-adhesive resin cements during the initial 24 h and after 5,000 thermocycles, no statistically significant differences were observed between the two modes after 10,000 thermocycles (Table 4). Therefore, it is recommended that the self-adhesive system, which is more time-efficient and has lower technique sensitivity, is ideal for cementation between zirconia restoration and titanium implant abutment.

These findings suggest that while initial bond strength may favor more technique-sensitive self-etching systems, self-adhesive cements offer sufficient long-term performance combined with procedural efficiency. Therefore, in clinical settings where time and ease of application are critical, self-adhesive cements present a viable alternative, particularly for luting zirconia crowns onto titanium abutments.

Fracture mode analysis provided additional insight into the nature of the bond failure. In the experimental group, most RU samples exhibited adhesive failure (score 1), transitioning to cohesive failure within the cement (score 2) after thermocycling. This shift indicates that thermocycling altered the weakest point from the interface to the cement bulk, reflecting partial degradation or internal stress distribution changes. The AI group showed a predominance of cohesive failures even prior to aging, indicating that the bonding interface was initially strong but the cement matrix was more susceptible to internal failure. These findings underscore the importance of both interface adhesion and internal cement properties in maintaining durable bonds.

The limitations of this in vitro study included the absence of oral environmental conditions, such as low pH challenges or masticatory stress. Additionally, the specific interactions between resin cements and zirconia–titanium interfaces warrant further investigation under more clinically realistic conditions.

## Conclusions

This study evaluated the SBS of six resin cements for bonding zirconia to titanium under simulated oral conditions. Self-etching cements (RU, AI) exhibited high initial SBS but significant decline after aging, whereas self-adhesive cements (IS, GM) demonstrated stable long-term performance. The unfilled MMA-based cement (SB) exhibited a unique increase in post-aging strength due to self-curing properties. Primer use (GMP) enhanced initial SBS but became negligible after thermocycling, suggesting optional primer application for efficiency.

Within the limitations of the current study and based on the findings, the following can be concluded: (1) SBS was significantly influenced by both bonding agent type and storage condition; (2) Bonding systems containing HEMA and 10-MDP exhibited high initial strength but declined significantly after thermocycling; (3) IS, GM, and GMP demonstrated stable bond durability.

## Data Availability

The datasets used and/or analyzed during the current study are available from the corresponding author on reasonable request.
